# Highly Efficient Amplification of Chronic Wasting Disease Agent by Protein Misfolding Cyclic Amplification with Beads (PMCAb)

**DOI:** 10.1371/journal.pone.0035383

**Published:** 2012-04-13

**Authors:** Chad J. Johnson, Judd M. Aiken, Debbie McKenzie, Michael D. Samuel, Joel A. Pedersen

**Affiliations:** 1 Environmental Chemistry and Technology Program and Department of Soil Science, University of Wisconsin, Madison, Wisconsin, United States of America; 2 Center for Prions and Protein Misfolding Diseases, University of Alberta, Edmonton, Alberta, Canada; 3 U.S. Geological Survey, Wisconsin Cooperative Wildlife Research Unit, University of Wisconsin, Madison, Wisconsin, United States of America; University of Maryland, United States of America

## Abstract

Protein misfolding cyclic amplification (PMCA) has emerged as an important technique for detecting low levels of pathogenic prion protein in biological samples. The method exploits the ability of the pathogenic prion protein to convert the normal prion protein to a proteinase K-resistant conformation. Inclusion of Teflon® beads in the PMCA reaction (PMCAb) has been previously shown to increase the sensitivity and robustness of detection for the 263 K and SSLOW strains of hamster-adapted prions. Here, we demonstrate that PMCAb with saponin dramatically increases the sensitivity of detection for chronic wasting disease (CWD) agent without compromising the specificity of the assay (i.e., no false positive results). Addition of Teflon® beads increased the robustness of the PMCA reaction, resulting in a decrease in the variability of PMCA results. Three rounds of serial PMCAb allowed detection of CWD agent from a 6.7×10^−13^ dilution of 10% brain homogenate (1.3 fg of source brain). Titration of the same brain homogenate in transgenic mice expressing cervid prion protein (Tg(CerPrP)1536^+/−^ mice) allowed detection of CWD agent from the 10^−6^ dilution of 10% brain homogenate. PMCAb is, thus, more sensitive than bioassay in transgenic mice by a factor exceeding 10^5^. Additionally, we are able to amplify CWD agent from brain tissue and lymph nodes of CWD-positive white-tailed deer having *Prnp* alleles associated with reduced disease susceptibility.

## Introduction

Transmissible spongiform encephalopathies (TSEs) or prion diseases are a class of rare progressive neurodegenerative diseases and include scrapie in sheep, bovine spongiform encephalopathy in cattle, and chronic wasting disease (CWD) in deer, elk and moose, and Creutzfeldt-Jakob disease in humans. The etiological agents in TSEs lack specific nucleic acid and are referred to as prions. Substantial experimental evidence indicates that prions are composed primarily, if not solely, of a disease-associated conformer of the host-encoded prion protein (PrP) [1], [2]. The central biochemical event in the propagation of prion diseases is the refolding of the normal cellular prion protein (PrP^C^) into a disease-associated conformer (PrP^TSE^), a substantial fraction of which partially resists digestion by proteinase K (PK) and is denoted PrP^res^. The precise molecular mechanism of PrP^C^-to-PrP^TSE^ conversion is unknown. Template-assisted refolding and nucleation-polymerization models have been advanced as mechanisms [3], [4]. The multiple stable conformations of PrP^TSE^ lead to the phenomenon of a single protein being able to encipher multiple strains.

Protein misfolding cyclic amplification (PMCA) [5] has emerged as an important technique for detecting low levels of PrP^res^ in biological samples [6]–[8]. PMCA exploits the ability of PrP^TSE^ to catalyze the conversion of PrP^C^ to the misfolded isoform in a manner conceptually similar to the polymerase chain reaction. The amount of PrP^res^ is increased by successive cycles of sonication (to disrupt PrP^TSE^ aggregates) and amplification using PrP^C^ from brain homogenate as a substrate. Sensitivity can be increased by replenishing the substrate after a round of PMCA (one round consists of multiple sonication-amplification cycles). The level of amplification is typically determined by immunoblotting of the amplification products following PK digestion. Sensitivity depends on the efficiency of amplification and the number of PMCA rounds. The lowest detection limit reported for infected brain homogenate (BH) is a 1×10^−12^ dilution of 10% BH from Syrian hamsters infected with the 263 K strain of hamster-adapted scrapie agent [9] carried through seven rounds of PMCA. This was estimated to be equivalent to ∼26 molecules or 1.3 ag of PrP^TSE^ [9], a thousand-fold more sensitive than the next most sensitive method (animal bioassay; 2×10^−9^ dilution of 10% BH or 5.3 fg). The PrP^res^ generated by PMCA is infectious [10] and retains the strain properties of the original PrP^TSE^ seed [11]. Adaptation of agent into a new species using PMCA faithfully mimics adaptation *in vivo* [12].

Optimal amplification conditions for PMCA vary by the prion strain being amplified [10], [12], [13]. Conditions for highly sensitive amplification of hamster-adapted scrapie have been determined [10]. For CWD agent (PrP^CWD^), continued improvement in sensitivity has been reported over the past several years [12], [14]. Kurt *et al.* (2007) demonstrated detection of PrP^CWD^ in a 6.5×10^−9^ dilution of 10% BH, gaining 200-fold increase in sensitivity with each round of serial PMCA. Meyerett et al. (2008) reported a 6,666-fold increase in detection with a single-round of PMCA, but did not report a limit of detection for PrP^CWD^ from a BH dilution series. Although we are able to achieve amplification of PrP^CWD^ under reported conditions [15], amplification was inconsistent, with rounds of PMCA intermittently failing to amplify any PrP^CWD^. Such inconsistencies in amplification have been noted previously [16]. This lack of robustness for PMCA negatively impacts the usefulness of the technique. Attempts to remedy these inconsistencies, such as addition of polyA RNA, have improved the robustness of the technique, but at the expense of increasing spontaneous generation of PrP^res^ in the absence of a PrP^TSE^ seed [17], [18]. This production of false positives precludes using the technique to screen samples in which minute levels of disease agent are expected. Recently, Gonzalez-Montalban et al. (2011) altered the PMCA format by including Teflon® beads (PMCAb) and observed increased robustness, rate, and yield of prion protein conversion, improving the sensitivity of detection for hamster-adapted TSE strains. Pritzkow et al. (2011) reported that inclusion of glass beads in the PMCA reaction also increases sensitivity and robustness for detection of the hamster-adapted 263 K prion strain and quantitatively linked seeding activity in PMCAb to infectivity *in vivo* [19]. In both PMCAb studies, the increases in robustness were not accompanied by a loss in specificity (i.e., no false positives), suggesting PMCAb may provide sensitive and robust amplification of other prion strains.

In this contribution, we demonstrate that PMCAb with Teflon® beads increases the rate and yield of PrP^CWD^ amplification, while maintaining high specificity. The sensitivity of PrP^CWD^ detection achieved after three rounds of serial PMCAb was higher than bioassay in transgenic cervidized mice (Tg(CerPrP)1536^+/−^ mice) by a factor of 10^5^. No false positives were generated with this method through six rounds of PMCAb. The robustness of amplification with beads was also markedly improved. The striking improvement in amplification efficiency and robustness achievable by PMCAb will aid CWD diagnostics, analysis of CWD disease progression and transmission as well as identification of environmental reservoirs of infectivity.

## Results

### Teflon® beads increase amplification rate and yield in PMCA of PrP^CWD^

**Figure 1:**
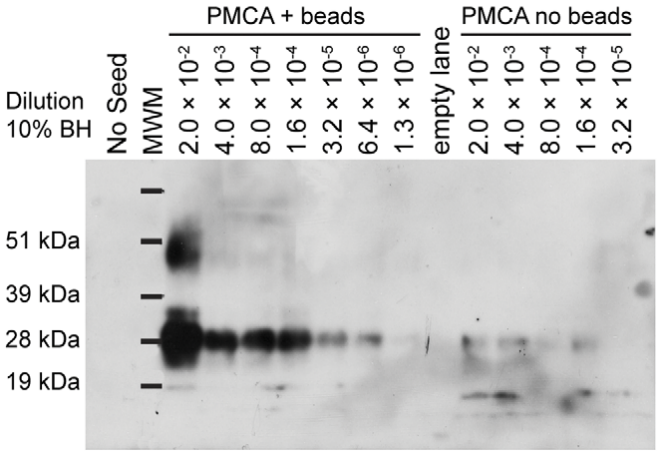
Teflon® beads in PMCA reaction increase sensitivity and yield of PrP^CWD^. CWD-positive brain homogenate (BH; 10% w/v) was serially diluted in normal BH (NBH). A 10 µL aliquot of the seed dilution was transferred into 90 µL NBH with 0.05% saponin in five-fold increments and subjected to PMCA (96 cycles) in the presence or absence of two 2.32 mm Teflon® beads (PMCAb). The no-seed control was 100 µL NBH treated as the other samples. MWM, molecular weight marker.

We evaluated the effect of Teflon® beads (two 2.32 mm beads) on the yield and rate of CWD agent amplification using the PMCA reaction conditions reported by Gonzalez-Montalban et al. (2011). PMCAb generated detectable levels of PrP^res^ in one 96-cycle round through the 1.3×10^−6^ dilution (2.5 ng brain) compared to 1.6×10^−4^ (312.5 ng source brain) in the no-bead control dilution series (**Fig. 1**). The primary source of PrP^CWD^ was brain tissue from clinically affected CWD-positive wt/wt white-tailed deer inoculated by the oral route (the wt/wt *Prnp* genotype is homozygous for amino acids Q and G at residues 95 and 96, respectively) [20]. Dilution series were prepared from 10% BH. Not only did inclusion of Teflon® beads boost the sensitivity of CWD prion detection by a factor of 125, but densitometric analysis indicated the yield of PrP^res^ in the 2×10^−2^ dilution was boosted by a factor of 7.3 when beads were included. Inclusion of Teflon® beads also improved the consistency of amplification results: the highest dilution detected agreed within a factor of five in five replicate single-round PMCAb experiments with Teflon® beads. For these experiments, 0.05% saponin was included in the normal brain homogenate (NBH).

### Saponin further increases the rate of PMCAb amplification

**Figure 2:**
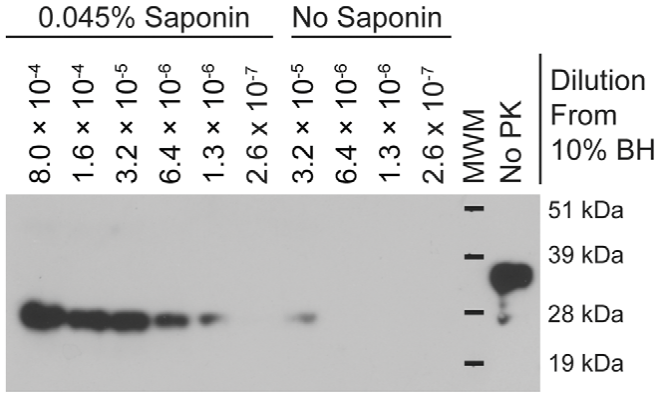
Saponin increases the sensitivity of PMCAb for PrP^CWD^ detection. CWD-positive brain homogenate (BH; 10% w/v) was diluted using serial five-fold dilutions in NBH and subjected to one round of 96-cycle PMCAb in the presence of absence of 0.045% saponin. BH, brain homogenate; MWM, molecular weight marker.

Inclusion of the amphipathic glycosides saponin or digitonin (0.05%) has been reported to increase the sensitivity of PMCA for PrP^RML^ [13] and PrP^CWD^ [15]. To quantitatively assess the effect of saponin on PMCAb of PrP^CWD^, a series of five-fold dilutions of the 10% BH was made in NBH lacking saponin. Aliquots (10 µL) of each dilution were added to 90 µL NBH with or without 0.05% saponin (0.045% final concentration). Samples were subjected to a single 96-cycle round of PMCAb. Sample location in the sonicator was changed each time the experiment was repeated to eliminate potential bias introduced by the position of sample tubes in the sonicator horn. Following PMCAb, 20 µL of each sample was fractionated by SDS-PAGE and immunoblotted. Three replicate experiments demonstrated that inclusion of saponin increased the sensitivity of PMCAb by a factor of five to 25 (**Fig. 2**). Interestingly, in the lower dilutions of CWD-positive BH, the amount of PrP^res^ produced was similar between the two dilution series with and without saponin, indicating that the saponin does not influence the upper limit of PrP^res^ produced, but rather the rate of production (data not shown). We also tested 0.05% digitonin in PMCAb amplification, but did not observe an increase in detection over saponin (data not shown). To determine whether the effect of saponin might be attributable to signal enhancement during Western blotting rather than to improved amplification, we spiked saponin or an equivalent amount of phosphate buffered saline into a dilution series that had undergone one round of PMCAb without saponin. No increase in detection was observed in the samples spiked with saponin (data not shown).

### Serial PMCAb is more sensitive than bioassay in Tg(CerPrP) mice

To determine the amount of infectious agent in the initial brain homogenate and to facilitate comparison of the sensitivity of PMCAb to bioassay, we titered the inoculum (serial 10-fold dilutions of 10% BH in PBS) in transgenic mice expressing cervid prion protein (Tg(CerPrP)1536^+/−^ mice) [21] via the intracerebral (i.c.) route of exposure. For the 10^−7^ dilution of BH, mice were euthanized after one year in the absence of signs of clinical disease, and four of eight mice exhibited PrP^res^ in brain tissue (**Supporting Fig. S1**). All mice (8/8) receiving the 10^−6^ dilution of CWD-positive BH exhibited clinical signs of ataxia, head tremors and, in some cases, mild kyphosis. Time to onset of clinical signs was 487±42 days for the 10^−6^ dilution of the inoculum. Based on this bioassay, log titer was estimated using the Kärber equation *M = χ_r_−d*(*S*′−0.5), where *M* is the estimated ID_50_, *χ_r_* is the highest dilution at which all mice succumbed to disease, *d* is the log-dose interval (10 in this case), and *S′* is the sum of positive frequencies from the last dilution that is 100% positive through to the highest dilution. Because the animals receiving the highest dilution were euthanized prior to manifestation of clinical symptoms, we excluded them from the calculation yielding a conservative estimate of 6.5 log i.c. ID_50_·30 µL^−1^ 10% BH or 9.0 log i.c. ID_50_·g^−1^.

**Figure 3:**
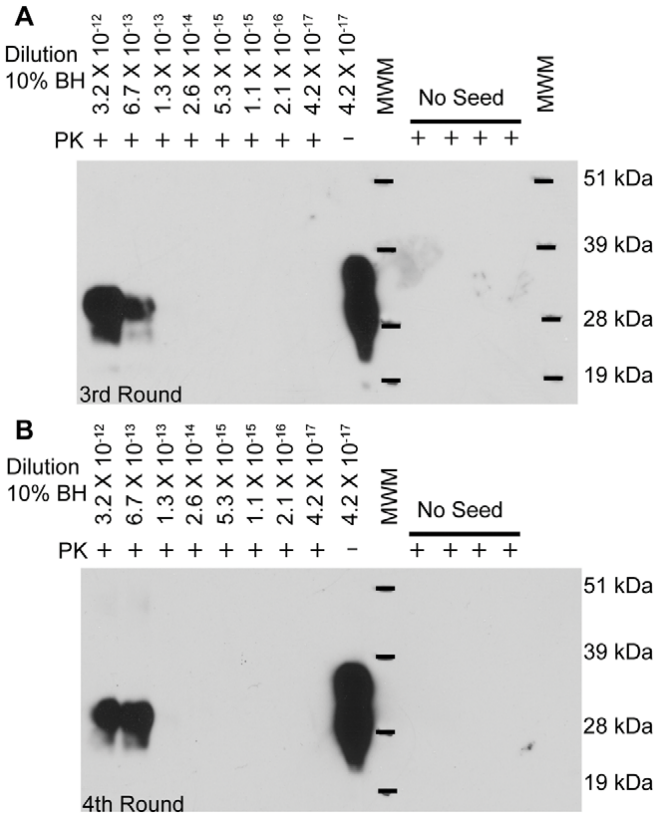
Serial PMCAb allows detection of PrP^CWD^ from femtogram levels of infected brain. CWD-positive BH (10% w/v) was diluted using serial five-fold dilutions in NBH and subjected to serial PMCAb in the presence of 0.05% saponin for four 96-cycle rounds. Results from the (A) third and (B) fourth rounds are shown. No-seed controls contained 100 µL NBH. MWM, molecular weight marker.

To compare the sensitivity achievable by PMCAb to that of bioassay, serial 5-fold dilutions of CWD-positive 10% BH in NBH were prepared, generating 2×10^−2^ to 4.2×10^−17^ dilutions. Each dilution was then subjected to serial PMCAb. After three rounds of serial PMCAb, PrP^res^ was detectable in the 6.7×10^−13^ dilution of 10% BH (1.3 fg of source brain) (**Fig. 3a**) with no further gain in sensitivity with a fourth round of PMCAb, as indicated by no signal in higher dilutions (**Fig. 3b**). The sensitivity of PMCAb in three serial 96-cycle rounds was thus higher than that of bioassay in cervidized Tg mice by a factor exceeding 10^5^.

**Figure 4:**
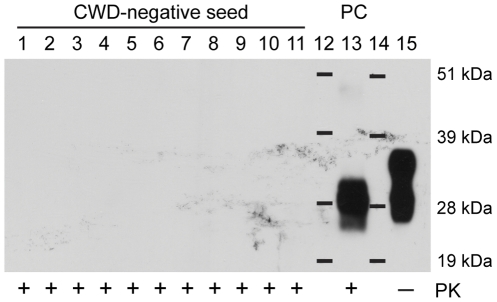
Serial PMCAb has high specificity. Eleven 10 µL CWD-negative white-tailed deer, homozygous for the wild-type *Prnp*, gene brain homogenate (BH; 10% w/v) samples (lanes 1–11) or 10 µL 3.2×10^−12^ dilution of wt/wt CWD+ white-tailed deer BH (lane 13) was added to 90 µL NBH with 0.05% saponin and subjected to six successive rounds of 96-cycle PMCAb. Samples from the sixth round were digested with proteinase K (PK; 50 µg·mL^−1^ final concentration) for 1 h at 37°C except one no-PK control.

High PMCAb sensitivity prompted concern that PrP^res^ might be generated spontaneously, producing false positive results. To investigate this possibility, we seeded 11 samples with CWD-negative deer BH (10% w/v) and performed six 96-cycle rounds of serial PMCAb. No PrP^res^ was detectable by immunoblotting after six rounds of PMCAb (**Fig. 4**). A positive control was carried through the six rounds of PMCAb to verify that amplification conditions were achieved. For all no-seed controls performed, no PrP^res^ was detected, this included 18 single-round, seven two-round, five three-round, and four four-round PMCAb reactions.

### Effect of bead material on amplification of PrP^CWD^

The composition of the beads used in PMCAb can affect amplification efficiency, and the optimal bead material appears to vary by prion strain [16]. We tested the effect of bead material on the amplification of PrP^CWD^. Polymeric bead materials, previously shown to enhance amplification of rodent-adapted prions, (Teflon®, EPDM, acetal, and nylon; [16]) as well as aluminum oxide (Al_2_O_3_) were tested. Glass and steel beads were not tested because they did not facilitate PrP^res^ amplification of rodent prion strains under similar PMCAb conditions [16], although in a different system smaller glass beads aided amplification [19]. CWD-positive 10% BH was diluted 6,250-fold in NBH and subjected to PMCAb using each bead material, or a no-bead control, for one 96-cycle round. Aluminum oxide, nylon and Teflon® all promoted amplification, while the other bead materials did not (**Supporting Fig. S2**). Teflon® beads resulted in the strongest enhancement of amplification.

### Kinetics of CWD prion amplification by PMCAb

**Figure 5:**
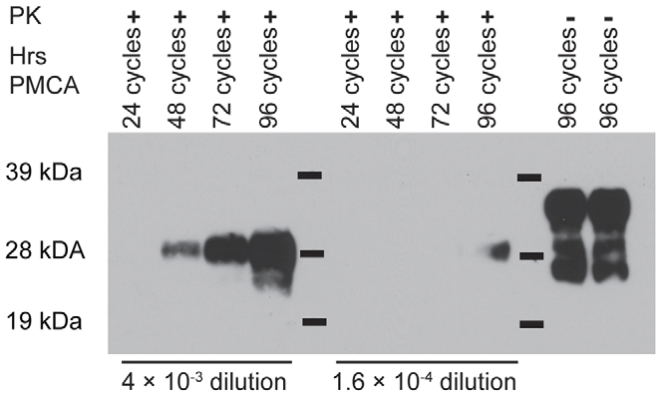
Kinetics of CWD amplification using PMCAb. Four 10 µL samples of 4×10^−3^ or 1.6×10^−4^ dilutions of CWD-positive BH (10%) were added to 90 µL NBH containing 0.05% saponin and subjected to PMCAb for the indicated number of cycles.

The number of cycles per PMCAb round impacts the amount of PrP^res^ generated in each round. Maximal amplification of the 263 K strain of hamster-adapted scrapie was achieved using 48-cycle PMCA rounds [16]. Using these conditions, we observed only a 5- to 25-fold increase in PrP^res^ per round in PMCAb of PrP^CWD^ (data not shown). To further explore the kinetics of PrP^res^ production in PMCAb amplification of PrP^CWD^, we prepared four replicate 4×10^−3^ and 1.6×10^−4^ dilution PMCAb reactions and removed one tube from each dilution after 24, 48, 72 and 96 cycles of PMCAb (**Fig. 5**). Densitometric measurement of the bands on the resulting immunoblots (ImageJ software) indicated that the band intensity for the 4×10^−3^ dilution was below detection at 24 cycles and the subsequent bands on the immunoblot increased in intensity with cycle number. For the 1.6×10^−4^ dilution, PMCAb did not produce detectable PrP^res^ until 96 cycles with band intensity similar to that of the 48-cycle band of the 4×10^−3^ dilution. If we use the 48-cycle band of the 4×10^−3^ dilution as a baseline, PrP^res^ accumulation increased by a factor of three by cycle 72 (an 0.125-fold average increase in intensity per cycle for the 24 cycles) and by a factor of 5.5 between cycles 48 and 96 (a 0.076-fold average increase per cycle over the last 24 cycles). The intensity of the cycle 96 band of the 4×10^−3^ dilution was 6.2 times higher than that of the cycle 96 band of the 1.6×10^−4^ dilution. If amplification rates were the same for the two dilutions, the band intensity would be expected to be 25-fold higher.

### Amplification of PrP^CWD^ from white-tailed deer differing in *Prnp* genotype and in non-brain tissues

**Figure 6:**
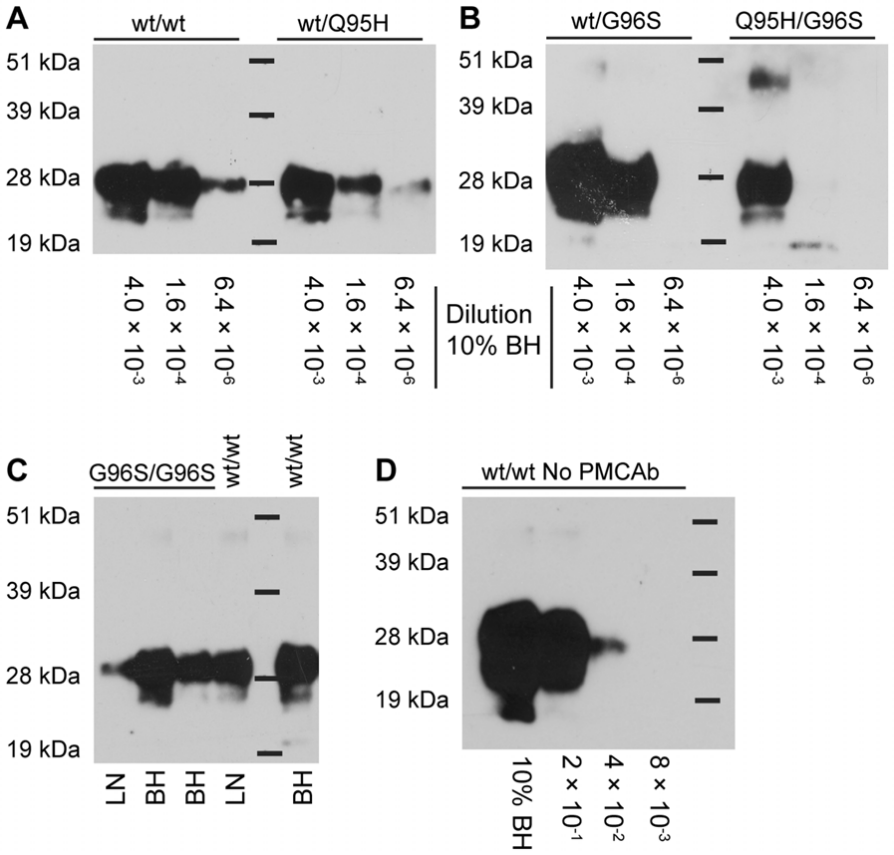
PMCAb amplifies PrP^CWD^ from white-tailed deer with allelic variations and from non-CNS tissues. Brain homogenates (BH; 10% w/v) from CWD-positive deer with allelic variations of *Prnp* were tested for their ability to seed PMCAb. CWD-positive 10% BH from (A) wt/wt or wt/95H deer and (B) wt/96S or 95H/96S white-tailed deer were serially diluted in normal BH (NBH) from Tg(CerPrP)1536^+/−^ mice, and 10 µL of the 25-, 625- and 1.6×10^4^-fold dilutions from each were used to seed 90 µL fresh NBH and subjected to one 96-cycle PMCAb round. (C) For testing the homozygous 96S allelic variant, retropharyngeal lymph nodes (LN) or BH from two separate hunter-harvested CWD-positive deer were used to seed NBH for PMCAb. As controls, homogenates of lymph nodes from a hunter-harvested CWD-positive wt/wt deer or orally inoculated wt/wt CWD clinically affected deer was used to seed a 96-cycle round of PMCAb. (D) Immunoblotting three five-fold dilutions of 10% BH from the end-stage wt/wt deer used for these studies after proteinase K digestion demonstrates that the PrP^CWD^ concentrations used in the PMCAb reactions (Fig. 6A) were not detectable without amplification.

Nucleotide polymorphisms in cervid *Prnp* alleles are associated with differential susceptibility to disease transmission and rates of disease progression in infected animals [20], [22]–[27]. In white-tailed deer, 95H and 96S alleles are associated with reduced susceptibility to CWD and slower disease progression in infected animals [16], [26]. We examined whether PrP^C^ from Tg(CerPrP)1536^+/−^ mice, which express the wt PrP allele, could support amplification of PrP^CWD^ from CWD-positive white-tailed deer expressing at least one copy of a resistance-associated allele. Serial five-fold dilutions were made in NBH of wt/wt, wt/95H, wt/96S, and 95H/96S CWD-positive deer brains from clinically affected animals [20], and 10 µL aliquots of the 4×10^−3^, 1.6×10^−4^ and 6.3×10^−6^ dilutions were used to seed 90 µL fresh NBH. Samples underwent 96 cycles of PMCAb, reaction mixtures were treated with PK (50 µg•mL^−1^), and PrP^res^ was detected by immunoblotting. NBH from Tg(CerPrP)1536^+/−^ mice supported amplification from all four allelic combinations (**Fig. 6**) with all three dilutions amplifying to detectable levels in the wt/wt and wt/95H seeded reactions. The wt/96S- and 95H/96S-seeded reactions also supported amplification. We obtained brain tissue from two hunter-harvested CWD-positive white-tailed deer homozygous for the 96S allele and prepared 10% (w/v) BH in PMCA buffer. We also prepared 5% homogenates of lymph nodes from hunter-harvested 96S homozygous deer and a wt/wt deer, both CWD-positive. After one 96-cycle round of PMCAb, PrP^res^ was detectable in all samples. (**Fig. 6B**).

## Discussion

The present study demonstrates that inclusion of Teflon® beads and saponin in the PMCA reaction dramatically improves the sensitivity and robustness of PrP^CWD^ detection. The sensitivity achieved by these modifications makes PMCAb detection of CWD agent comparable to that reported for the 263K and Hyper strains of hamster-adapted prions [9], [16]. Attaining amplification of levels of PrP^CWD^<1 i.c. ID_50_ in two rounds and at the limit of immunoblotting detection after three rounds of PMCAb minimizes opportunities for cross-contamination by reducing sample handling and markedly reducing the time of analysis when attempting to detect minute levels of CWD agent.

The improved sensitivity and robustness of PMCAb for CWD agent does not compromise the specificity of the assay. The method has demonstrated high specificity; no false positives have been produced, even when performing six rounds of amplification in serial PMCAb. This specificity is consistent with that observed for hamster-adapted prion strains [16], and suggests that PMCAb will be useful in detecting prions from multiple species and in environmental matrices. With one exception, the PMCAb reactions reported here used PrP^CWD^ derived from brain tissue. Infectious agent derived from other tissues or environmental matrices may contain compounds that promote non-specific conversion of PrP^C^ to PrP^res^ or inhibit PMCA. Confident application of PMCAb to diverse sample types requires rigorous assessment of this possibility.

Teflon® beads and the amphipathic glycoside saponin appear to have an additive effect on CWD agent amplification, with the influence of the beads being stronger. At present, the mechanisms by which these components increase amplification are unknown. Teflon® beads have been shown to facilitate the fragmentation of recombinant prion protein fibrils [16]. The optimal bead material for PMCAb varies by prion strain and the number of beads does not substantially affect conversion efficiency, suggesting that physical agitation of the reaction mixture by the beads does not provide an adequate explanation for the mechanism by which beads facilitate fragmentation of PrP fibrils. The effect of bead material on energy distribution in the reaction vessel or on the adsorption of PrP or cofactors may warrant investigation. The generally low affinity of biomolecules for Teflon®, however, argues against the importance of the latter. Saponin disrupts cholesterol-rich lipid rafts, thus promoting conversion efficiency by increasing the dispersion of PrP^C^ [13], [28].

The source of PrP^C^ for PMCAb conversion in our study was BH from perfused Tg(CerPrP)1536^+/−^ mice [21]. This mouse line has been used extensively for both *in vivo* and *in vitro* studies of CWD, making it ideal for characterizing our CWD agent source. We titered the CWD agent used in this study in Tg(CerPrP)1536^+/−^ mice and determined that 30 µL of the 10^−7^ dilution of 10% (w/v) BH transmitted biochemical hallmarks of disease to half of the mice inoculated. We note that the 10^−7^ dilution represented the highest dilution used. If the presence of PrP^res^ in these mice is sufficient evidence for infection, then 0.3 ng of clinically affected CWD-positive white-tailed deer brain contains ∼1 ID_50_ as assayed in Tg(CerPrP)1536^+/−^ mice. This corresponds to 9.5 log i.c. ID_50_•g^−1^ and agrees with Kärber method for estimation of titer if the 10^−7^ dilution is included, exceeding the levels of infectivity previously reported for other CWD agent sources characterized in this mouse line [29], [30]. The highest previously reported titer for CWD was 8.5 log i.c. ID_50_·g^−1^ from a mule deer inoculum into a different cervidized mouse line, Tg(ElkPrP) mice [31]. At least two factors may have contributed to this difference. First, the CWD agent used in the present study derived from experimentally infected white-tailed deer housed in animal care facilities, which may have facilitated longer survival times with higher accumulation of disease agent. Second, the CWD agent present in Wisconsin white-tailed deer may result in higher accumulations of PrP^CWD^ compared to the levels accumulated in CWD-positive mule deer.

The sensitivity of serial PMCAb was higher than that of bioassay in Tg(CerPrP)1536^+/−^ mice: serial PMCAb allowed detection of the 6.7×10^−13^ dilution of 10% CWD-positive BH after three rounds while biochemical hallmarks of disease were detected in half of the mice inoculated at the 10^−7^ dilution. Thousand-fold increases in sensitivity over bioassay have been reported for serial PMCA of hamster-adapted prions [9], but the >10^5^ increase is unprecedented.

Our study extends the utility of PMCAb to a new prion strain and demonstrates that this method is substantially more sensitive than animal bioassay. The higher sensitivity and increased robustness of PMCAb is expected to allow early stages of disease to be delineated (i.e., PrP^TSE^ trafficking during initial exposure), determination of efficacies of decontamination methods, and detection of CWD agent in naturally contaminated environmental samples. Enabling the identification of environmental reservoirs of infectivity would lead to better understanding of CWD epizootics.

## Materials and Methods

### Ethics Statement

All animals were cared for in accordance with protocols approved by the Institutional Animal Care and Use Committee of the University of Wisconsin, Madison (Assurance Number A3464-01).

### Preparation of CWD-infected tissue homogenates

CWD-infected BH (10% w/v) in phosphate buffered saline (PBS) was prepared from whole brain of orally inoculated clinically affected white-tailed deer (*Odocoileus virginianus*). The CWD-positive deer had the following Prnp genotypes: wt/wt, wt/95H, 95H/96S, or wt/96S [20]. Although most experiments were performed using the wt/wt CWD-positive BH, tissue homogenates also were prepared from hunter-harvested, immunohistochemistry-positive 96S/96S white-tailed deer retropharyngeal lymph node (5% w/v) and obex (10% w/v) in PMCA buffer (described below) or wt/wt white-tailed deer lymph node (5% w/v).

### Determining CWD agent titer in transgenic (CerPrP) mice

Brain homogenate (10% w/v) from a CWD-positive wt/wt white-tailed deer brain was briefly sonicated (10 s) at 70% intensity in a cup horn sonicator and serially diluted in PBS to produce inocula ranging from 10% to 0.000001% w/v BH. Ten transgenic mice (27–35 days old) expressing cervid prion protein (Tg(CerPrP)1536^+/−^ mice [21]) per dilution were intracerebrally inoculated with 30 µL inoculum and monitored weekly for disease symptoms. Upon manifestation of early symptoms (slight ataxia in the hind limbs), mice were monitored three times weekly for disease progression. Mice were euthanized when pronounced ataxia and head tremors were noted, but before notable weight loss. In a few mice, mild kyphosis developed. Tg(CerPrP)1536^+/−^ mice are prone to tumors and only eight mice survived to manifest clinical symptoms in each cohort except the 0.1% dilution in which all 10 mice survived to the clinical stage.

### Protein misfolding cyclic amplification (PMCA)

Uninfected Tg(CerPrP)1536^+/−^ mice, age 4–10 months, were euthanized by CO_2_ asphyxiation and immediately perfused with modified Dulbecco's phosphate buffered saline without Ca^2+^ or Mg^2+^ (Thermo Scientific #SH30028.02) amended with 5 mM EDTA. Brains were then rapidly removed, flash frozen in liquid nitrogen, and stored at −80°C until use. Brains were homogenized on ice to 10% w/v using a Wheaton Potter-Elvehjem homogenizer in PMCA conversion buffer (Fisher Sci. #0841414C). The conversion buffer consisted of Ca^2+^- and Mg^2+^-free DPBS supplemented with 150 mM NaCl, 1% Triton X100, 0.05% saponin (Mallinckrodt #H277-57), 5 mM EDTA, and 1 tablet Roche Complete EDTA-free protease inhibitors cocktail (Fisher Sci. # 50-720-4069) per 50 mL conversion buffer. In some experiments, the saponin was omitted or replaced with 0.05% digitonin (Sigma # D-5628). Brain homogenates were clarified by centrifugation (2 min, 2,000 *g*). Supernatant was transferred to pre-chilled microcentrifuge tubes, flash frozen in liquid nitrogen, and stored at −80°C until use.

Seeds for PMCA and PMCAb were prepared from clinically affected CWD-positive white-tailed deer by 1∶1 dilution of 20% (w/v) BH in PBS with the conversion buffer described above. The resulting 10% BH was serially diluted five-fold in normal brain homogenate to generate dilution series ranging from 2×10^−2^ to 4.2×10^−17^. Seeds for PMCAb from hunter-harvested obex and lymph nodes were made by homogenizing tissues in 1∶1 mixture of DPBS and conversion buffer described above, to a final concentration of 10% (w/v).

The dilutions (10 µL) were used to seed 90 µL NBH in 0.2 mL thin-walled PCR tubes with (PMCAb) or without (PMCA) two 2.38 mm beads. The majority of PMCAb experiments employed Teflon® beads (McMaster-Carr, #9660K12). Beads made of other materials were tested for their effect on conversion efficiency: nylon (#B000FMUECC), acetal (ball grade 1, #B000FN0OP8), EPDM (#B00137UR7A), and aluminum oxide (grade 10, #BAO09310) (Small Parts, www.smallparts.com). Experimental tubes were placed in a rack in a Misonix S-3000 or S-4000 microplate horn, and the reservoir was filled with 300 mL water. PMCA and PMCAb conditions consisted of 96 cycles in a Misonix S-3000 or Misonix S-4000. Each cycle consisted of 30 s sonication at 50% to 70% of maximum power followed by 29 min 30 sec incubation at 37°C. With time, individual sonicators required adjustments in sonication energy delivered. For the Misonix S-4000, the optimal power for CWD amplification was 125–135 W. For serial PMCAb, at the conclusion of one 96-cycle round of PMCAb, 10 µL of each dilution was transferred to 90 µL fresh NBH and subjected to another 96-cycle round of PMCAb.

### Detection of PrP^res^ by immunoblotting

We prepared PMCA samples for immunoblotting by a method adapted from Haley *et al.*, (2009). Briefly, 20 µL of each PMCA reaction was transferred to a thin-walled 96-well plate and mixed with 3 µL PK (500 µg mL^−1^) and 7 µL dilution buffer (0.1% [v/v] Triton-X 100, and 4% (w/v) SDS in PBS) and incubated for 1 h at 37°C with shaking (500 rpm). Following PK digestion, 10 µL 4× LDS sample buffer (Invitrogen, #NP0008) containing 200 mM dl-1,4-dithiothreitol (Fisher Scientific, #AC16568-0050) was added, and the sample was incubated for 25 min at 99°C with shaking (600 rpm). Proteins in 16 or 28 µL samples (and in 4 and 5 µL no-PK controls) were fractionated on 15-well 10% Bis-Tris Gels (Invitrogen, #NP0303BOX) or 12-well 12% Bis-Tris Gels (Invitrogen, #NP0342BOX), respectively, and electrotransferred to polyvinylidene fluoride (PVDF) membranes (Millipore, #IPVH00010). The PVDF membranes were blocked for 1 h in 3% (w/v) powdered nonfat-milk in TBST prior to application of mAb 8G8 (1∶1,000 in 3% milk TBST; Cayman Chemicals, #189760). Membranes were rinsed with TBST, and horseradish peroxidase conjugated goat anti-mouse IgG (Bio-Rad #170-5047) 1∶20,000 dilution in 5% milk TBST was applied. After rinsing the membranes with TBST, ECL Plus immunoblot detection system (GE Healthcare, #RPN2132) or Supersignal West Pico chemiluminescent substrate (Thermo Scientific, #34080) was used to detect immunoreactivity.

### Densitometry

ImageJ software was used for densitometric analysis. Blots were scanned at 300 dpi and saved as *.tif files. The densitometric analysis was conducted following the procedure of Miller (www.lukemiller.org/ImageJ_gel_analysis) and represented an adaptation of the procedure outlined in the imageJ documentation (http://rsb.info.nih.gov/ij/docs/menus/analye.html#gels). No protein standards were used in these analyses; an internal band was used as the standard, and other bands expressed as fold changes from that band.

## Supporting Information

Figure S1 **PrP^CWD^ present in brains of Tg(CerPrP)1536^+/−^ mice inoculated intracerebrally with a 10^7^ dilution of 10% w/v CWD-positive BH. **Mice were euthanized prior to manifestation of clinical symptoms, and their brains were assayed for PrP^res^ by Western blotting. Samples were treated with PK, a 20 µL aliquot of each sample was resolved on a 12-well 12% Bis-Tris gel and visualized by Western blotting using mAb 8G8 and goat anti-mouse HRP conjugated secondary Ab. Lane 1 is an uninfected control Tg(CerPrP)1536^+/−^ mouse, lane 3 is from a clinically positive Tg(CerPrP)1536^+/−^ mouse from the 10^2^ dilution of CWD-positive 10% BH and lanes 5–12 are from the Tg(CerPrP)1536^+/−^ mice receiving the 10^7^ dilution of CWD-positive 10% BH.
(TIF)

Figure S2 **Teflon beads allow the largest amplification of CWD associated PrP^res^ during PMCAb.** Teflon beads were tested against beads of other material (all 2.38 mm) in one round of PMCAb consisting of 96 cycles. CWD+ brain homogenate (BH; 10%) was diluted 6,250-fold in normal BH (NBH), and a 10 µL aliquot was added to 90 µL fresh NBH in tubes containing no beads, or two EPDM, aluminum oxide, acetal, nylon or Teflon beads. After PMCAb, samples were digested with proteinase K (PK; 50 µg•mL^−1^ final concentration) for 1 h at 37°C. A 28 µL aliquot of each sample (5 µL for the no PK control) were resolved on a 12 well 12% Bis-Tris gel and visualized by immunoblotting using mAb 8G8 and goat anti-mouse HRP conjugated secondary Ab. MWM, molecular weight marker.
(TIF)
