# Supplementary figures and images for: Highly Efficient Amplification of Chronic Wasting Disease Agent by Protein Misfolding Cyclic Amplification with Beads (PMCAb)
Source: PLoS One. 2012 Apr 13;7(4):e35383. doi: 10.1371/journal.pone.0035383 (PMC3325955; doi:10.1371/journal.pone.0035383)

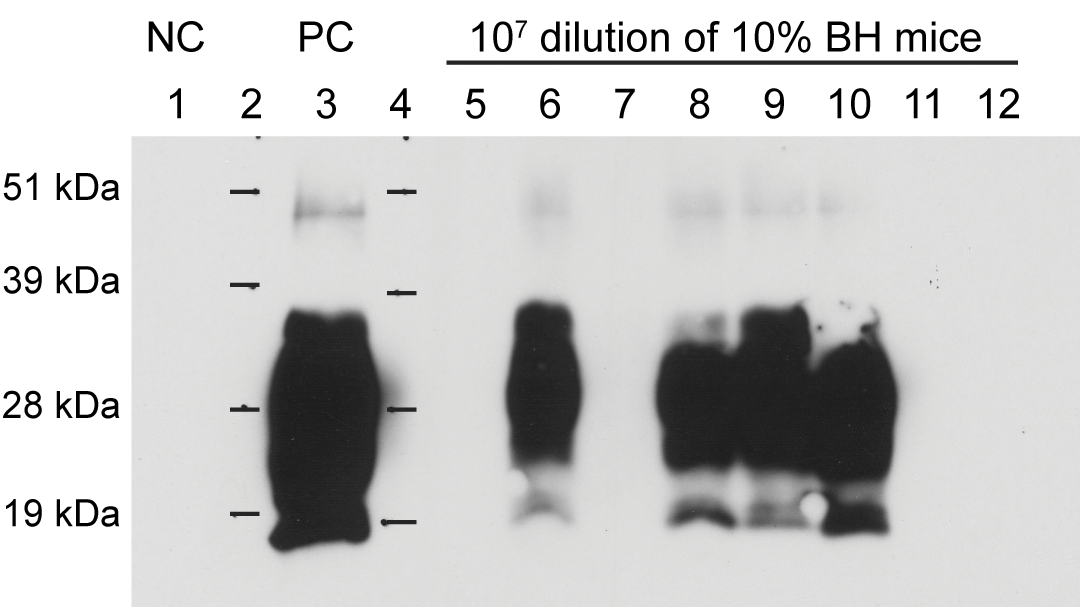

Supplement: Figure S1 — PrPCWD present in brains of Tg(CerPrP)1536+/− mice inoculated intracerebrally with a 107 dilution of 10% w/v CWD-positive BH. Mice were euthanized prior to manifestation of clinical symptoms, and their brains were assayed for PrPres by Western blotting. Samples were treated with PK, a 20 µL aliquot of each sample was resolved on a 12-well 12% Bis-Tris gel and visualized by Western blotting using mAb 8G8 and goat anti-mouse HRP conjugated secondary Ab. Lane 1 is an uninfected control Tg(CerPrP)1536+/− mouse, lane 3 is from a clinically positive Tg(CerPrP)1536+/− mouse from the 102 dilution of CWD-positive 10% BH and lanes 5–12 are from the Tg(CerPrP)1536+/− mice receiving the 107 dilution of CWD-positive 10% BH. (TIF) [file pone.0035383.s001.tif]

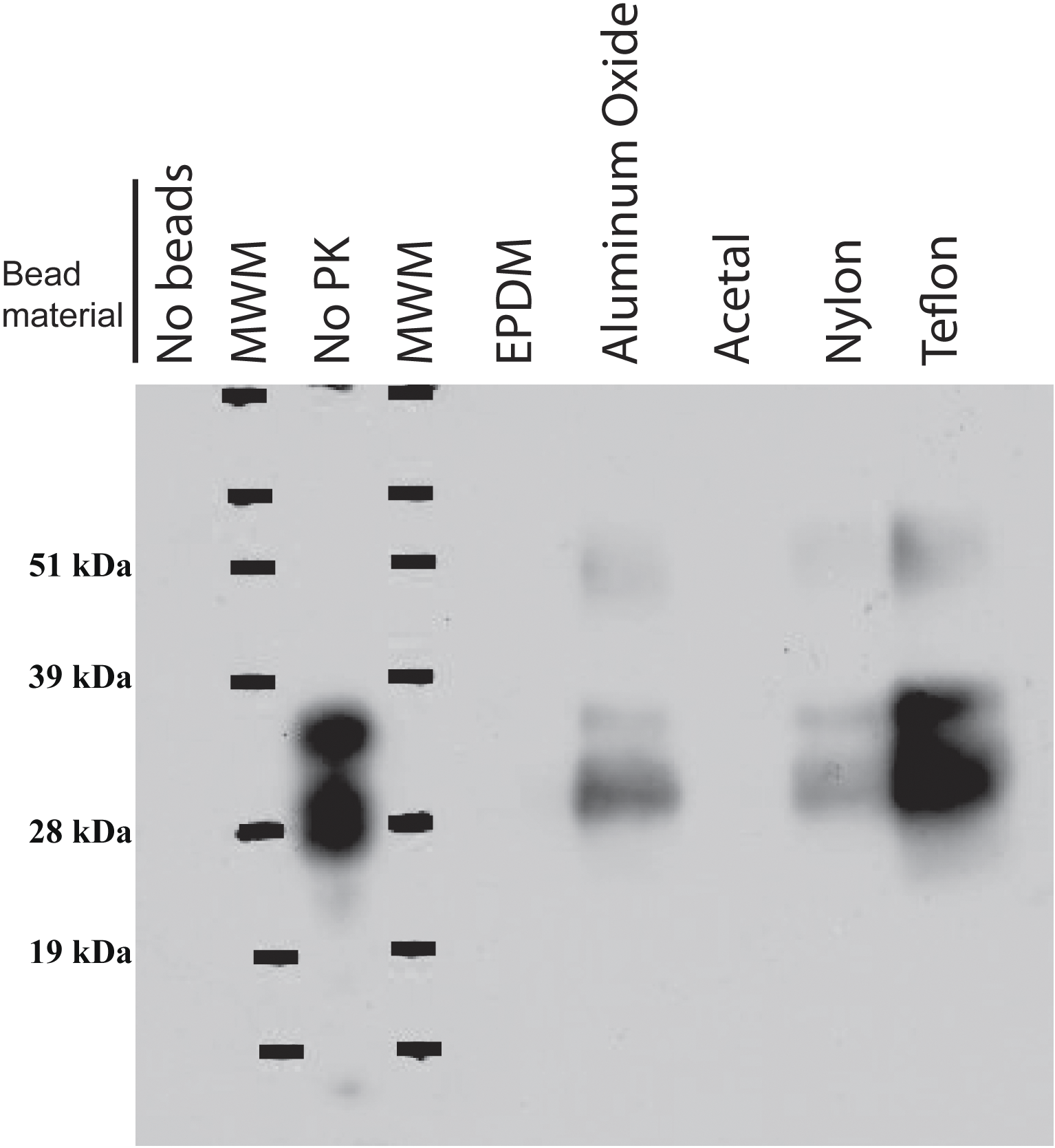

Supplement: Figure S2 — Teflon beads allow the largest amplification of CWD associated PrPres during PMCAb. Teflon beads were tested against beads of other material (all 2.38 mm) in one round of PMCAb consisting of 96 cycles. CWD+ brain homogenate (BH; 10%) was diluted 6,250-fold in normal BH (NBH), and a 10 µL aliquot was added to 90 µL fresh NBH in tubes containing no beads, or two EPDM, aluminum oxide, acetal, nylon or Teflon beads. After PMCAb, samples were digested with proteinase K (PK; 50 µg•mL−1 final concentration) for 1 h at 37°C. A 28 µL aliquot of each sample (5 µL for the no PK control) were resolved on a 12 well 12% Bis-Tris gel and visualized by immunoblotting using mAb 8G8 and goat anti-mouse HRP conjugated secondary Ab. MWM, molecular weight marker. (TIF) [file pone.0035383.s002.tif]
